# Toxin-neutralizing antibodies elicited by naturally acquired cutaneous anthrax are elevated following severe disease and appear to target conformational epitopes

**DOI:** 10.1371/journal.pone.0230782

**Published:** 2020-04-15

**Authors:** Eric K. Dumas, Hayati Demiraslan, Rebecca J. Ingram, Rebecca M. Sparks, Emily Muns, Adriana Zamora, Jason Larabee, Lori Garman, Jimmy D. Ballard, Geert-Jan Boons, Judith A. James, Uner Kayabas, Mehmet Doganay, A. Darise Farris

**Affiliations:** 1 Arthritis and Clinical Immunology Program, Oklahoma Medical Research Foundation, Oklahoma City, Oklahoma, United States of America; 2 Department of Microbiology and Immunology, University of Oklahoma Health Sciences Center, Oklahoma City, Oklahoma, United States of America; 3 Department of Infectious Diseases, Faculty of Medicine, Erciyes University, Kayseri, Turkey; 4 Centre for Infection and Immunity, Queen’s University Belfast, Belfast, Northern Ireland, United Kingdom; 5 Complex Carbohydrate Research Center and Department of Chemistry, University of Georgia, Athens, Georgia, United States of America; 6 Department of Chemical Biology and Drug Discovery, Utrecht Institute for Pharmaceutical Sciences and Bijvoet Center for Biomolecular Research, Utrecht University, Utrecht, The Netherlands; 7 Departments of Medicine and Pathology, University of Oklahoma Health Sciences Center, Oklahoma City, Oklahoma, United States of America; 8 Department of Infectious Diseases and Clinical Microbiology, Faculty of Medicine, Inonu University, Malatya, Turkey; University at Albany, SUNY, UNITED STATES

## Abstract

Understanding immune responses to native antigens in response to natural infections can lead to improved approaches to vaccination. This study sought to characterize the humoral immune response to anthrax toxin components, capsule and spore antigens in individuals (n = 46) from the Kayseri and Malatya regions of Turkey who had recovered from mild or severe forms of cutaneous anthrax infection, compared to regional healthy controls (n = 20). IgG antibodies to each toxin component, the poly-γ-D-glutamic acid capsule, the *Bacillus* collagen-like protein of *anthracis* (BclA) spore antigen, and the spore carbohydrate anthrose, were detected in the cases, with anthrax toxin neutralization and responses to Protective Antigen (PA) and Lethal Factor (LF) being higher following severe forms of the disease. Significant correlative relationships among responses to PA, LF, Edema Factor (EF) and capsule were observed among the cases. Though some regional control sera exhibited binding to a subset of the tested antigens, these samples did not neutralize anthrax toxins and lacked correlative relationships among antigen binding specificities observed in the cases. Comparison of serum binding to overlapping decapeptides covering the entire length of PA, LF and EF proteins in 26 cases compared to 8 regional controls revealed that anthrax toxin-neutralizing antibody responses elicited following natural cutaneous anthrax infection are directed to conformational epitopes. These studies support the concept of vaccination approaches that preserve conformational epitopes.

## Introduction

*Bacillus anthracis* is a Gram-positive, spore-forming bacterium that is the causative agent of anthrax infection. While anthrax is naturally a disease of animals, humans can also be incidentally infected. Human infection occurs through contact with *B*. *anthracis* spores, commonly through the handling of contaminated animal materials. Infection in humans can also occur through the deliberate release of spores as a biological weapon, as was evidenced in the United States (US) in 2001 [1, 2]. There are currently four main classifications of anthrax based on the route of infection: cutaneous anthrax, gastrointestinal anthrax, inhalational anthrax, and injectional anthrax [3]. Cutaneous anthrax is initiated when *B*. *anthracis* spores penetrate the skin, often through a cut or abrasion. While 95% of anthrax cases worldwide are cutaneous, this classification also has the lowest mortality rate, ranging from 10 to 40% without treatment to <1% with antibiotic therapy [4, 5].

Cutaneous anthrax is diagnosed from history of exposure to sick animals or animal products and clinical picture of cutaneous anthrax, which includes a characteristic skin lesion at the site of spore inoculation. A painless, pruritic, papular lesion appears after an approximate 5-day incubation period then evolves to a vesicle with central necrosis and drying to produce a characteristic black eschar [2]. Even with antibiotic therapy, the disease can follow a mild or severe course distinguished by lesion size, presence or absence of bullae and edema, as well as presence or absence of systemic symptoms [5]. Uncommon but life-threatening complications include toxemic shock, extensive edema and anthrax meningitis [5, 6]. Though effective public animal health programs, including veterinary vaccination, have limited zoonotic and collateral human disease outbreaks in many locations globally [2], cutaneous anthrax remains common in several countries including Turkey [4].

*B*. *anthracis* has two major virulence factors, a poly-γ-D-glutamic acid capsule and a secreted tripartite toxin [7]. The capsule plays an anti-phagocytic role, allowing the bacteria to evade engulfment by macrophages [8]. The tripartite toxin is made up of three secreted proteins, protective antigen (PA), lethal factor (LF) and edema factor (EF) [9]. These three proteins combine to form two AB toxins, lethal toxin (LT, a combination of PA and LF) and edema toxin (ET, a combination of PA and EF) [9, 10]. PA, an 83kD protein, serves as the common host-cell binding component of both toxins. PA binds to one of the two major anthrax toxin receptors and forms an endosomal pore, allowing LF and EF access to the cytosol, where they are able to exert their effects [11]. LF is a 90kD Zn^2+^-dependent metalloprotease that cleaves MAPKKs, while EF is an 89kD calmodulin-dependent adenylate cyclase that converts ATP to cAMP [12, 13]. These toxins act to impair both the innate and adaptive immune systems, and have further effects during late, systemic disease [14, 15].

Evaluation of immune responses to spontaneous infection [16–19], as well as to existing vaccines, can provide knowledge useful for improving approaches to vaccination. For example, we previously identified several protective and non-protective antibody specificities to toxin antigens in anthrax vaccine recipients [20–22]. The observation that the Anthrax Vaccine Precipitated preparation contains all three anthrax toxin components, yet lacks toxin activity [23], suggests that important neutralizing epitopes could be lost due to a change in structure or conformation of anthrax toxin components by alum precipitation. Thus, assessment of the fine specificity of the toxin-neutralizing response to native toxin antigens encountered during natural infection may reveal new toxin-neutralizing epitopes.

The purpose of the present study was to determine the fine specificity and toxin neutralization capacity of the humoral immune response to natural cutaneous infection and to compare humoral immune responses in different forms of the disease. Sera from recovered cutaneous anthrax patients from the Kayseri and Malatya regions of Turkey contained antibodies directed to each toxin component, the poly-γ-D-glutamic acid capsule, the *Bacillus* collagen like protein of *anthracis* (BclA), and the spore carbohydrate antigen anthrose. Antibody levels and toxin neutralization capacity were significantly higher in individuals who had recovered from severe compared to mild forms of disease. Comparison of PA, LF and EF overlapping decapeptide binding patterns in cutaneous cases compared to regional healthy controls showed that anthrax toxin-neutralizing antibodies retained following natural cutaneous infection are likely directed to conformational epitopes.

## Materials and methods

### Collection of human serum samples

Blood samples were collected from individuals in the Malatya (n = 21) or Kayseri (n = 25) regions of Turkey who had been previously treated for cutaneous anthrax in the years spanning 2003–2012. Regional healthy controls (n = 20) were recruited from the Kayseri region in 2012. Sera were isolated from whole blood via centrifugation, frozen and shipped to the US on dry ice. Upon thawing, aliquots were made and re-frozen. Some aliquots were supplemented with sodium azide for preservation. Total freeze/thaw cycles were limited to two.

### Ethics statement

All blood samples from individuals who had recovered from cutaneous anthrax infection, as well as from healthy regional Turkish controls (n = 20) with no prior history of anthrax infection, were collected by informed consent under the oversight of the clinical ethics committee of Erciyes University. Use of sera isolated from these samples, as well as pre-existing serum samples from 120 healthy controls from the United States (US), was approved by the Institutional Review Board of the Oklahoma Medical Research Foundation. Collection and use of all samples and clinical data followed guidelines established by the Declaration of Helsinki. All adult subjects provided written informed consent, and a parent or guardian of any child participant provided written informed consent on their behalf.

### Disease categorization

The patients were categorized into mild or severe groups by medical record as previously described [5], with mild disease having lesions < 4 cm in diameter surrounded by narrow erythema and lack of systemic symptoms and severe disease having lesions > 4 cm in diameter, presence of bullae and extensive edema. The two patients with toxemic shock, defined as the presence of a cutaneous lesion along with systemic symptoms including fever, rapid heart rate, rapid breathing, acute mental, changes, hypotension and negative blood culture, were included in the severe cutaneous anthrax group for the purposes of statistical analysis.

### Antibody quantification by ELISA

PA, LF and EF ELISAs were performed using commercially available antigens (List Biologicals, Campbell, CA) as previously described [21]. Cutaneous anthrax and regional control group sera were titered using 2-fold dilutions, beginning at 1:10. US control sera were titered at 10-fold dilutions, beginning at 1:10. Endpoint titers were defined as the last dilution giving an optical density (OD) greater than the average OD plus 2 standard deviation (SD) above values of the control groups (tested at 1:80 dilution for regional Turkish controls; tested at 1:100 dilution for US controls). PA IgG concentration was calculated using a standard curve of reference serum AVR801 (Center for Disease Control and Prevention, Atlanta, GA) containing antibodies to PA, serially diluting 2-fold at a starting concentration of 109.4 μg/mL [24].

Anti-BclA ELISAs were performed as described [25], with minor modifications. Briefly, 96-well plates were coated with 100 μL per well of 1.25 μg/mL rBclA (BEI Resources) overnight at 4°C. The plates were blocked with PBS containing 0.1% BSA for 1 hour (h) at room temperature (RT), followed by a 2 h incubation with serum at RT. Alkaline-phosphatase conjugated anti-human secondary (Jackson ImmunoResearch Laboratories, West Grove, PA) was added to the plates and incubated for 2 h at RT, followed by the addition of substrate. OD_405_ was measured using a Biotek Hybrid Synergy H1 plate reader (Biotek, Winooski, VT). Endpoint titers were defined as the reciprocal of the last dilution with OD_405_ values exceeding the average OD_405_ plus 2SD of the region-matched healthy control samples at a 1:80 dilution.

Anti-capsule IgG was measured by standard ELISA. Briefly, 96-well plates were coated with 50 μg/ml (100 μl/well) of poly-γ-D-glutamic acid (Anaspec Inc., Fremont, CA) and incubated overnight at 4°C. Wells were washed 4 times with 1X PBS-Tween and blocked with 150 μl of 0.1% BSA/0.02% NaN_3_ in PBS at RT for 1 h. Wells were washed 4 times and serum samples diluted in 1X PBS-Tween with 0.1% BSA were added, followed by a 2 h incubation at RT. Wells were washed 4 times, and a 1:10,000 dilution of alkaline phosphatase-labeled anti-human IgG (Jackson ImmunoResearch, West Grove, PA) was added and incubated at RT for 2 h. Wells were washed and bound antibodies were detected with the addition of 75 μl of 1 mg/ml 4-nitrophenyl phosphate substrate (Sigma-Aldrich St. Louis, MO) at RT, and OD_410_ was measured using a Dynex Technologies Revelation 4.25 plate reader (Dynex Technologies, Chantilly, VA). Endpoint titers were defined as described for BclA.

A rhamnose/anthrose disaccharide-BSA conjugate was synthesized as described [26] and used in ELISAs as similarly described for an anthrose-containing trisaccharide [27], with minor modifications. Briefly, 96-well plates were coated with 100 μL per well of the rhamnose/anthrose-BSA disaccharide conjugate at a concentration of 0.03 μg/mL and incubated overnight at 4°C. Plates were blocked with a 5% skim milk in PBS-Tween for 1 h at RT. Serum samples were added and incubated for 2 h at RT. Horse-radish peroxidase conjugated anti-human secondary (KPL, Gaithersburg, MD) was added at a dilution of 1:20,000, followed by a 2 h incubation at RT. Bound antibodies were detected by the addition of SureBlue Reserve TMB Substrate (KPL). Substrate was incubated at RT for 8 minutes, and the reaction was stopped by the addition of 100 μL TMB stop solution (KPL). OD_450_ was measured, and endpoint titers were defined as described for BclA and capsule antigens.

### J774A.1 LT neutralization assay

This assay was performed as described [28], with minor modifications. J774A.1 cells from The European Collection of Authenticated Cell Cultures were plated at 90,000 cells per well in 96-well plates and incubated overnight at 37°C. The following day, serum serial dilutions (beginning undiluted, then serially diluted two-fold to create a 10 point dilution curve) were pre-incubated with 25 ng PA and 5 ng LF for 30 min at 37°C, then added to the cells and incubated for 3 hours (h). Next, 50 μL of 1.5 mg/mL Thiazolyl Blue Tetrazolium Bromide (MTT) was added, and the plates were incubated for 1 h. The media was then removed. The cells were solubilized for 30 min, followed by OD measurement and ED_50_ value calculation using four-parameter logistic regression [29].

### ET neutralization assay

Cyclic AMP production and ET neutralization were assessed using a RAW 264.7 cAMP response element (CRE) luciferase reporter line [30]. Cells (100,000/well) were cultured in 96-well plates for 18 h at 37°C. The cells were then treated with final concentrations of 0.25 μg/mL PA and 0.25 μg/mL EF (equivalent to 0.025 μg of each/well) either without (ET Only) or with sample (serum at a 1:10 dilution, or mAb), and incubated at 37°C for 4 h. Cells were then washed twice with PBS, lysed by the addition of 50 μL Passive Lysis Buffer (Promega, Madison, WI), then stored at -80°C. Luciferase expression levels were quantified using a luciferase assay system (Promega) followed by measurement of luminescence with LMax II 384 luminometer (Molecular Devices, Sunnyvale, CA). Luciferase expression in untreated wells was subtracted from all treatment wells to account for endogenous cAMP, and neutralization percentage was calculated as (ET Only ÷ [ET + sample]) X 100.

### Solid phase ELISAs

Decamer peptides overlapping by 8 amino acids and spanning the entire length of the PA (Genbank accession number AAA22637), LF (Genbank accession number AAM26117), EF (Genbank accession number AAA79215), and BclA (Genbank accession number CAD56869.1) proteins were covalently synthesized onto polyethylene sold phase supports in a 96-well format as previously described [31]. Peptides were incubated with a 1:200 dilution of serum for 2 h at RT, followed by washing and addition of peroxidase labeled goat anti-human IgG (KPL, Gaithersburg, MD) with an overnight incubation at 4°C. The following day, peptides were washed and bound antibodies were detected by the addition of SureBlue Reserve TMB substrate (KPL). An epitope was defined as one or more solid-phase peptides with an OD_450_ value greater than or equal to the average OD_450_ plus 2SD of a group of 8 region-matched control samples for each peptide, OD greater than 0.2, and recognized by more than 50% of all antibody positive samples.

### Statistical analysis

PA, LF and EF IgG ELISA between group comparisons were assessed by the Kruskal-Wallis test. All other ELISAs, toxin neutralization assays and severity comparisons were assessed by unpaired, 2-tailed Mann-Whitney U tests. In all comparisons, mean ± SEM is reported. Between-group proportions were compared by Fisher’s exact test, and associations were reported as odds ratios (OR). Correlations were analyzed by 2-tailed Spearman correlation tests. All statistical analyses were performed using GraphPad Prism 6.0.

## Results

### Clinical characteristics

Cutaneous anthrax was diagnosed in the patients (n = 46) by history of contact with ill animals or contaminated animal products and the presence of one or more lesions. Clinical characteristics and demographics of the patients are listed in Tables S1 and 1. Gram stain or bacterial culture confirmed the diagnosis in 18 cases. The site of the lesion or lesions for all patients was on the upper body, most often located on the finger, wrist or arm and less commonly on the face or neck. Of these 46 patients, 18 (18/46, 39%) had experienced a mild infection, and 28 (28/46, 61%) had experienced a severe clinical course. Three patients with lesions on the neck or face developed toxemic shock and/or extensive edema. Regardless of the severity of infection, all the patients recovered with antibiotic treatment. No differences in the time post-infection that the samples were collected (Mild group: median 46 months, range 4–102 months; Severe group: median 50 months, range 1–102 months), incubation time, duration of antibiotic therapy, age at infection or sex, between the mild and severe groups were noted (Table 1).

**Table 1:** Clinical and demographic characteristics of 46 cutaneous anthrax patients.

	Mild Cutaneous Anthrax (n = 18)^a^	Severe Cutaneous Anthrax (n = 28)^b^
**Time Post-Infection (Months)**		
Average (SEM)	46.3 (7.3)	48 (6.6)
Median	38.1	50.7
Range	4.3–102.2	1–102.2
**Incubation Time (Days)**^**b**^		
Average (SEM)	5.9 (0.3)	5.3 (0.8)
Median	5	4.5
Range	2–15	1–20
**Duration of Therapy (Days)**^**a**^		
Average (SEM)	5.1 (0.3)	6.7 (0.50)
Median	5	5
Range	3–7	4–14
**Age at Infection**		
Average (SD)	34.6 (16)	36.5 (12)
IQR	19.5–51.25	23–37
Range	19–64	23.75–48
**Sex**	67% Male	75% Male

^a^Duration of therapy was unknown for two patients, one with mild disease and one with severe disease.

^b^Incubation time was unknown for six patients with severe disease.

### Cutaneous anthrax infection elicits neutralizing antibodies to toxin components

**Fig 1:**
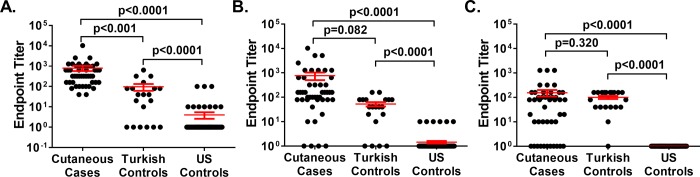
Serum IgG titers to anthrax toxin components in recovered cutaneous anthrax cases compared to controls. Endpoint titers of serum IgG to recombinant Protective Antigen (PA) **(A)**, Lethal Factor (LF) **(B)** and Edema Factor (EF) **(C)**, in individuals from Turkey who had recovered from cutaneous anthrax infection (n = 46), regional Turkish controls with no history of prior anthrax infection (n = 20) and US controls with no history of prior anthrax infection (n = 120). End-point titers were calculated as the last serum dilution exceeding a threshold of positive binding equivalent to 2SD above the mean of the US controls at a 1:100 dilution. Differences between groups were determined using the Kruskal-Wallis test with Dunn’s multiple comparisons post-test.

Serum samples from cutaneous anthrax survivors (n = 46), regional healthy controls with no known prior anthrax infection (n = 20), and US healthy controls with no known prior anthrax infection (n = 120) were tested for antibodies against PA, LF and EF. Mean antibody levels (PA) and titers (LF and EF) are shown in Fig 1 and Table 2. Using US controls to establish thresholds for positivity, the serum samples from cutaneous survivors were more likely to be positive (titer ≥ 80) for IgG to each toxin component (PA: 44/46 (95.7%), LF: 38/46 (82.6%), EF- 25/46 (54.3%)) than healthy control samples from the US (PA: 3/120 (2.5%), LF: 0/120 (0%), EF: 0/120 (0%)). Cutaneous survivors also had significantly higher antibody levels to each component compared to US controls. Increased antibody incidence (PA: 4/20 (20%), LF: 8/20 (40%), EF: 12/20 (60%)) and higher antibody levels to each component were also observed in the region-matched healthy controls compared to the US controls (Fig 1 and Table 2).

**Table 2:** Serum IgG levels to bacillus anthracis toxin components in cutaneous anthrax survivor and control groups. ^a^

	Cutaneous (n = 46)	US Controls (n = 120)	Turkish Controls (n = 20)
PA IgG (μg/mL, mean ± SEM)	799.6 ± 226.6	4.0 ± 15.6	96.8 ± 35.6
LF IgG (Titer, mean ± SEM)	767.9 ± 266.4	1.5 ± 2.0	52.7 ± 10.6
EF IgG (Titer, mean ± SEM)	154.2 ± 46.1	0	100.6 ± 14.2

^a^See Fig 1 for statistical comparisons.

**Fig 2:**
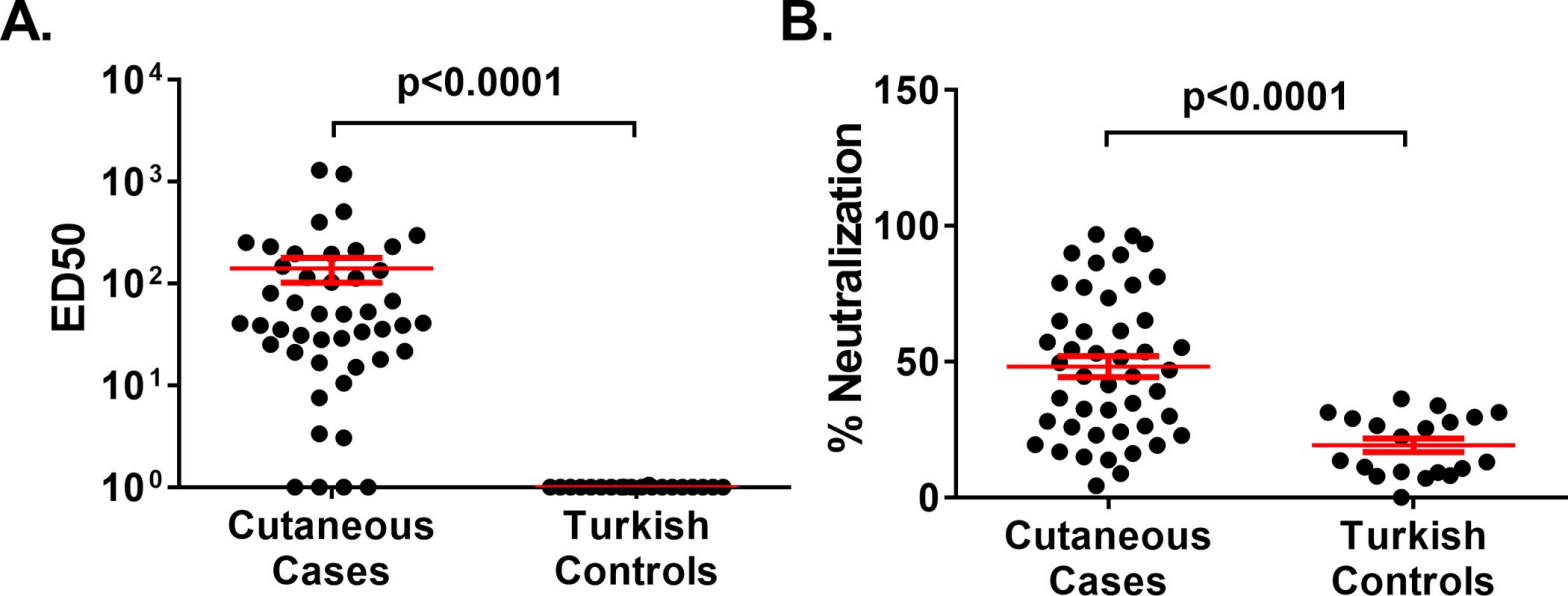
Anthrax toxin neutralization in recovered cutaneous anthrax cases compared to regional healthy controls. **(A)** 50% effective dilution (ED50) values for serum neutralization of Lethal Toxin (LT) determined in a J774A.1 macrophage-based LT neutralization assay using serum from cutaneous anthrax survivors (n = 46) compared to regional healthy controls with no history of prior anthrax infection (n = 20). **(B)** Edema Toxin (ET) neutralization by 1:10 dilutions of serum from cutaneous anthrax survivors (n = 46) compared to regional healthy controls with no history of prior anthrax infection (n = 20) using a cyclic AMP reporter macrophage cell line. Data are expressed as percentage of value obtained in the absence of serum or plasma antibodies. Red lines show mean ± SEM for all panels. P-values determined by Mann-Whitney U test.

While antibodies from the cutaneous anthrax survivors neutralized both LT (ED_50_: 140.6±38.5) and ET (% Neutralization at 1:10 serum dilution: 48.1±3.9), antibodies from the region-matched healthy controls failed to neutralize either toxin above baseline levels (LT ED_50_: 1.0±0.003; ET % Neutralization at 1:10 serum dilution: 19.2±2.5, values no different from that of 50 healthy US controls: 16.8±2.7) (Fig 2A and 2B). The observation that some of the Turkish control samples had magnitudes of IgG binding to toxin components overlapping that of the documented cases, but no toxin neutralizing activity, suggested that some binding activity to *B*. *anthracis* toxin components may be due to unknown cross-reactive environmental exposures common to the region or to subclinical exposures to *B*. *anthracis*. Therefore, only regional controls were used to establish thresholds of positivity for antibody titers in further tests.

### Spore and capsule specific antibodies are elicited during cutaneous anthrax infection

**Fig 3:**
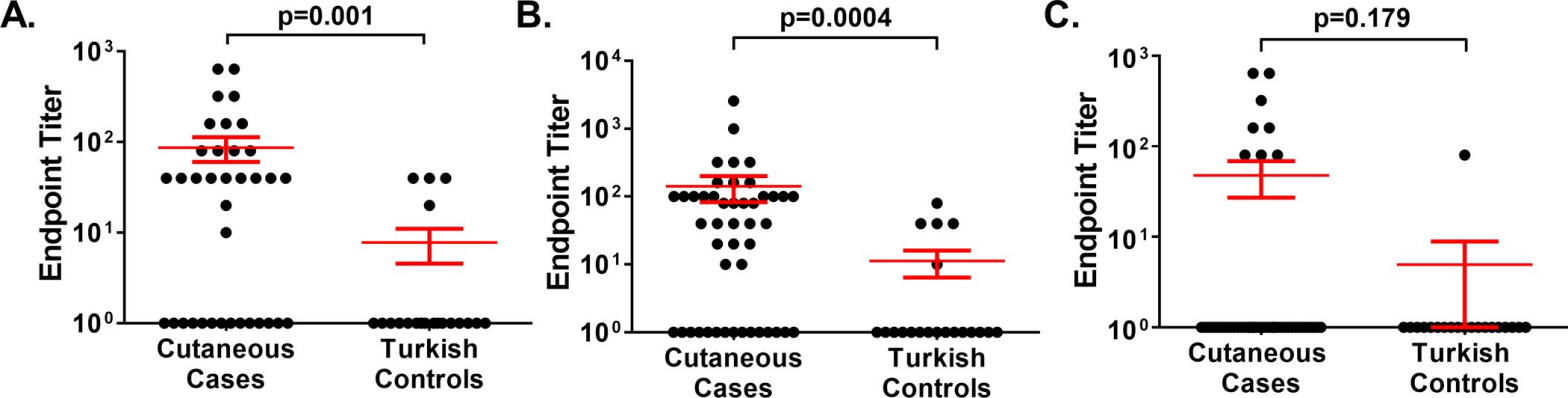
Serum IgG titers to capsule and spore antigens in cases compared to regional controls. Endpoint titers of serum IgG reactivity to synthetic poly-γ-D-glutamic acid capsule **(A)**, recombinant BclA **(B)** and synthetic anthrose disaccharide **(C)** antigens in sera from recovered cutaneous anthrax cases (n = 36 for panel (A); n = 46 for panels (B) and (C)) and in sera from regional Turkish controls (n = 20). End-point titers were calculated as the last serum dilution exceeding a threshold of positive binding equivalent to 2SD above the mean of the regional controls at a 1:80 dilution. Red lines show mean ± SEM for all panels. Differences between groups were determined by Mann-Whitney U test.

During natural cutaneous anthrax infection, the host is exposed to both the spore and encapsulated bacterial forms of *B*. *anthracis*. Therefore, samples from the cutaneous anthrax patients (n = 46) and regional controls (n = 20) were evaluated for IgG binding to the poly-γ-D-glutamic acid capsule antigen, as well as the spore components BclA and a synthetic rhamnose/anthrose disaccharide. The cutaneous anthrax serum samples were more likely (11/36, 30.6%) than the regional healthy controls (0/20, 0%) to contain anti-capsule IgG at an end titer ≥ 80 (p = 0.0048, OR 18.5) and had significantly elevated anti-capsule IgG titers (cutaneous anthrax: 73.4±21.1; regional controls: 7.8±3.2, p = 0.0012) (Fig 3A). The samples from the cutaneous anthrax survivor cohort were also more likely to have anti-BclA IgG (cutaneous anthrax end titer ≥ 80: 21/46, 45.7%; regional controls: 1/20, 5%; p = 0.0013, OR 16.0) at significantly elevated titers (cutaneous anthrax: 141.6±58.7) than the regional healthy controls (11.3±4.8, p = 0.0004, Fig 3B). In contrast, while some recovered cutaneous anthrax samples contained antibodies with anthrose binding activity, occurrence of binding at titers ≥ 80 (cutaneous anthrax: 8/46, 17.4%; Turkish controls 1/20, 5%; p = 0.2573) or magnitude of end-point titer (cutaneous anthrax: 47.8±20.7; Turkish Controls: 5.0±4.0, p = 0.179) did not significantly differ from the healthy regional controls (Fig 3C). To our knowledge, this is the first study investigating the presence of antibodies to BclA or anthrose antigens in recovered cutaneous anthrax samples.

### Correlative relationships among humoral responses to *B*. *anthracis* antigens observed in known cutaneous anthrax cases are absent in regional controls

As subclinical seroconversion to *B*. *anthracis* toxin antigens has been documented [32–35], reactivity to multiple *B*. *anthracis* antigens in the regional control group could provide evidence of seroconversion due to subclinical spore exposure. Thus, correlative relationships among the humoral IgG titers to the toxin components, capsule and spore antigens were determined in the confirmed cases and compared to that of the regional Turkish controls. Within the group of confirmed cases, positive correlative relationships were observed among IgG responses to PA and LF (r = 0.324, p = 0.028), PA and EF (r = 0.303, p = 0.041), LF and EF (r = 0.320, p = 0.013) (Table 3). A statistical correlation between IgG responses to LF and capsule (r = 0.401, p = 0.015) was also observed, though its biological significance, if any, is uncertain. These relationships were notably absent from the group of 20 regional controls (Table 4). Responses to anthrose and BclA spore antigens showed no correlative relationship in either cases or controls. Apparent correlation between responses to EF and anthrose detected in the control group (r = 0.498, p = 0.025) was driven exclusively by one sample showing positive binding to both anthrose (titer 80) and EF (titer 80). This analysis revealed a qualitatively dissimilar aspect of humoral reactivity to *B*. *anthracis* components among the regional healthy controls compared to those who had recovered from clinically apparent infection.

**Table 3:** Spearman correlation matrix of IgG ELISA titers to *B*. *anthracis* antigens in cutaneous anthrax cases (n = 46)^a, b^.

	PA	LF	EF	Capsule^b^	Anthrose	BclA
**PA**	r = 1.000	***r = 0.324***	***r = 0.303***	r = 0.241	r = -0.120	r = -0.066
		***p = 0.028***	***p = 0.041***	p = 0.157	p = 0.427	p = 0.665
**LF**		r = 1.000	***r = 0.364***	***r = 0.401***	r = 0.277	r = -0.051
			***p = 0.013***	***p = 0.015***	p = 0.063	p = 0.738
**EF**			r = 1.000	r = 0.320	r = -0.193	r = -0.273
				p = 0.057	p = 0.200	p = 0.067
**Capsule**				r = 1.000	r = 0.045	r = 0.128
					p = 0.793	p = 0.457
**Anthrose**					r = 1.000	r = -0.005
						p = 0.973
**BclA**						r = 1.000

^a^All p-values are from 2-tailed Spearman correlations. Significant values are in bold italics.

^b^IgG titers to capsule antigen were not available from 10 cases due to insufficient serum availability; therefore, correlations with IgG titers to capsule utilized data from 36 patients.

**Table 4:** Spearman correlation matrix of IgG ELISA titers to *B*. *anthracis* antigens in healthy controls from Turkey (n = 20)^a^.

	PA	LF	EF	Capsule	Anthrose	BclA
**PA**	r = 1.000	r = 0.001	r = -0.326	r = 0.014	r = -0.131	r = -0.065
		p = 0.995	p = 0.160	p = 0.953	p = 0.582	p = 0.785
**LF**		r = 1.000	r = 0.046	r = 0.407	r = -0.232	r = 0.094
			p = 0.847	p = 0.075	p = 0.326	p = 0.692
**EF**			r = 1.000	r = 0.044	***r = 0.498***^b^	r = 0.222
				p = 0.854	***p = 0.025***	p = 0.347
**Capsule**				r = 1.000	r = -0.114	r = 0.393
					p = 0.632	p = 0.086
**Anthrose**					r = 1.000	r = 0.393
						p = 0.086
**BclA**						r = 1.000

^a^All p-values are from 2-tailed Spearman correlations.

^b^Removal of one sample with positive IgG titers to EF (80) and anthrose (80) resulted in no correlation (r = 0). Significant values are in bold italics.

### Severity of infection affects the quality of the humoral immune response

**Fig 4:**
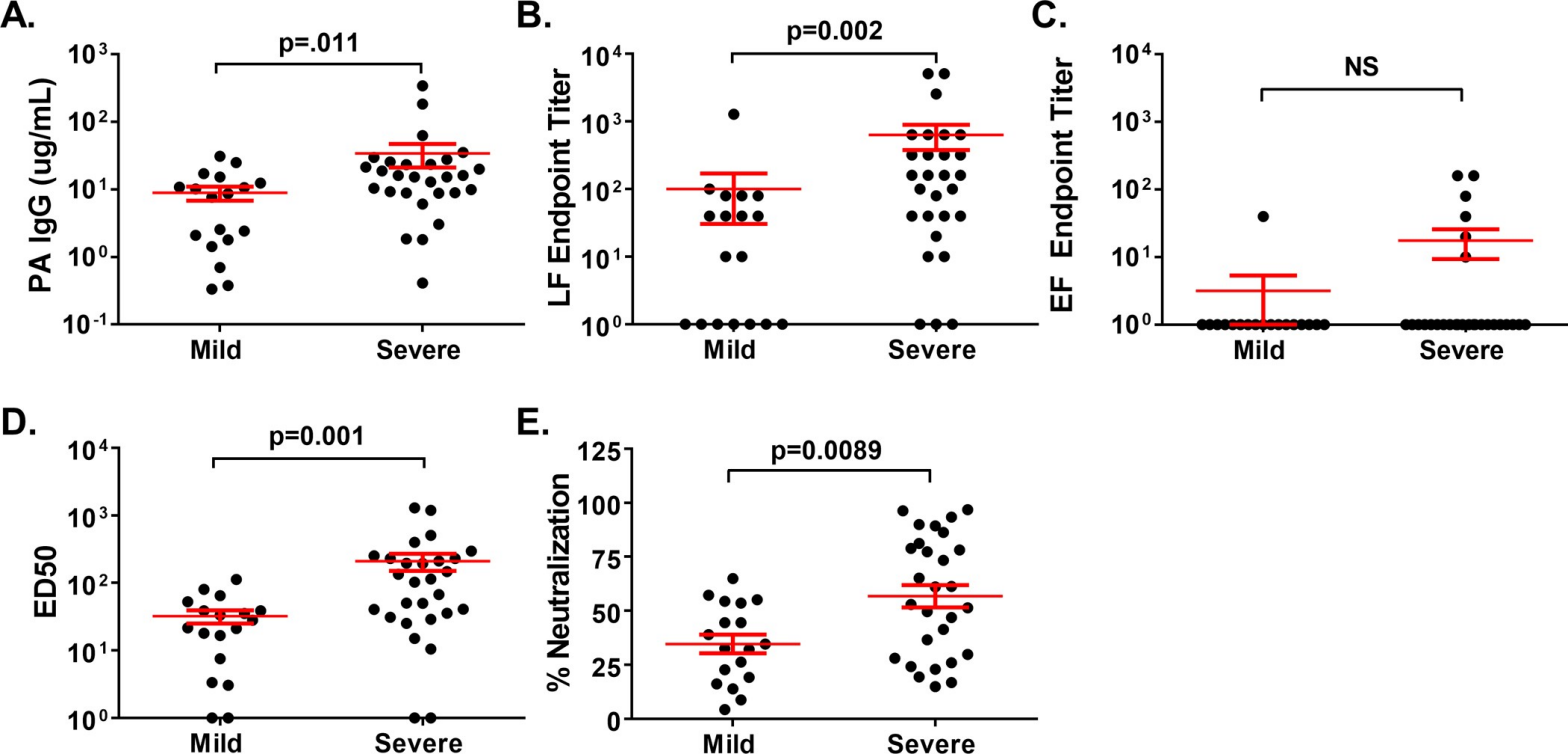
Effect of cutaneous anthrax disease severity on the humoral response to *B*. *anthracis* toxins. **(A)** Concentration of IgG binding to recombinant Protective Antigen (PA), **(B)** end-point titer of serum IgG to recombinant Lethal Factor (LF) and **(C)** end-point titer of serum IgG to recombinant Edema Factor (EF) in individuals who had recovered from mild (n = 18) or severe (n = 28) cutaneous anthrax infection. End-point titers for A-C were calculated as the last serum dilution exceeding a threshold of positive binding equivalent to 2SD above the mean of the regional controls. **(D)** 50% effective dilution (ED50) values for serum neutralization of Lethal Toxin (LT) determined in a J774A.1 macrophage-based LT neutralization assay in the same mild and severe cutaneous anthrax sera shown in A-C, and **(E)** Percent inhibition of Edema Toxin (ET) neutralization by 1:10 dilutions of sera from the same mild and severe cases evaluated in A-D using a cyclic AMP reporter macrophage cell line. Red lines show mean ± SEM for all panels. Differences between groups were determined by Mann-Whitney U test.

We then examined the impact of disease severity on the humoral immune response. Serum samples from severe cutaneous anthrax cases contained higher amounts of PA IgG (34.3±13.1 μg/mL) than those from mild cases (8.9±2.1 μg/mL, p = 0.011) (Fig 4A). Similarly, sera from severe cutaneous anthrax cases contained higher titers of LF IgG (634.4±256.9) than sera from mild cases (100.4±69.9, p = 0.002) (Fig 4B). However, no statistical differences in the magnitude of responses to EF (Fig 4C) or to capsule antigen, BclA or anthrose (p = 0.859, 0.534 and 0.333 by Mann-Whitney U test, respectively; S2 Table) due to disease severity were observed. Evaluation of the LT neutralization ED_50_ values by severity revealed that antibodies from severe cutaneous anthrax survivors had significantly higher LT neutralization (210.3±59.9) than antibodies from mild cutaneous anthrax survivors (32.2±7.1, p = 0.001) (Fig 4D). Similar results were obtained when ET neutralization percentages were examined, with the antibodies from severe cutaneous anthrax survivors having significantly higher capacity to neutralize ET (56.8±5.2) than antibodies from mild cutaneous anthrax survivors (34.7±4.3, p = 0.009) (Fig 4E). These results are unlikely to be due to demographic differences, as recovered cutaneous anthrax cases with mild (n = 18) or severe (n = 28) disease were similar (Table 1). Thus, severe cutaneous anthrax infection elicits more robust responses to anthrax toxin components.

### LT and ET neutralizing antibodies fail to bind linear peptide epitopes

**Fig 5:**
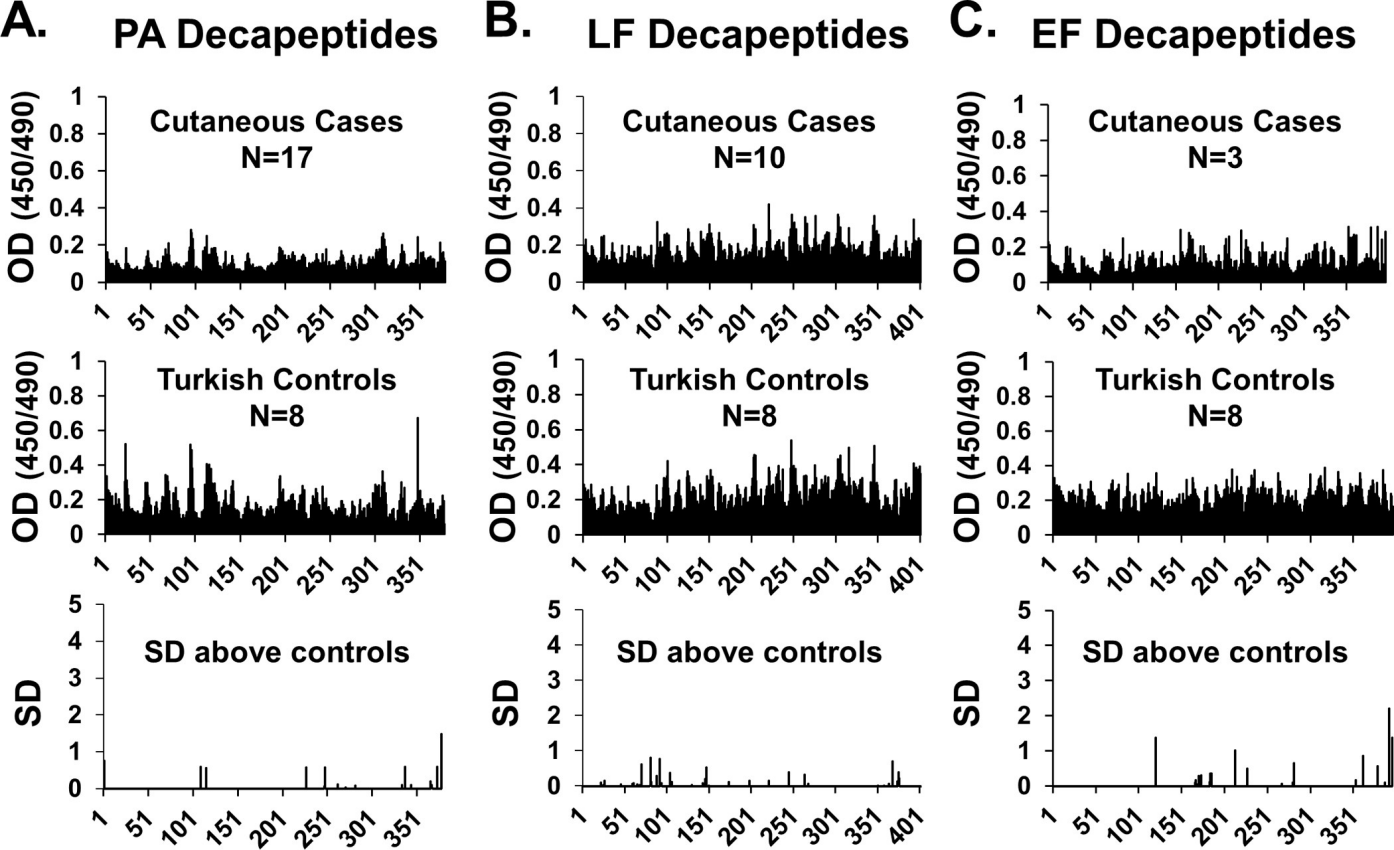
Binding of case and regional control serum IgG to overlapping decapeptides of toxin components. **(A)** Average IgG binding to overlapping decapeptides of Protective Antigen (PA) using serum samples from 17 recovered cutaneous anthrax cases with PA IgG titers ≥ 80 (top) and 8 regional Turkish controls (middle). The lower panel shows binding of sera from the 17 PA IgG positive cases above the mean of the 8 regional controls expressed as number of SD above the mean for each decapeptide. **(B)** Average IgG binding to overlapping decapeptides of Lethal Factor (LF) using serum samples from 10 recovered cutaneous anthrax cases with LF IgG titers ≥ 80 (top) and 8 regional Turkish controls (middle). The lower panel shows binding of the case samples above the control samples expressed as SD above the mean for each decapeptide. **(C)** Average IgG binding to overlapping decapeptides of Edema Factor (EF) using serum samples from 3 recovered cutaneous anthrax cases with EF IgG titers ≥ 40 (top) and 8 regional Turkish controls (middle). The lower panel shows case binding in terms of number of SD above the mean of control sera binding for each decapeptide. Thresholds of standard ELISA positivity used to include samples in the analysis shown were based on regional control data.

To determine the fine specificity of the LT- and ET-neutralizing response of patients, antibodies were tested against sequential overlapping epitopes of PA, LF and EF. From the initial cohort of 26 patients, those highly positive (titers ≥80) for PA IgG (n = 14) and LF IgG (n = 7) were used for PA and LF peptide binding experiments, respectively. For EF peptide binding experiments, two subjects whose samples were highly positive (titer≥80) and one subject whose sample was moderately positive (titer ≥40) were utilized. These three samples also exhibited high binding to PA and LF and were thus mapped on all three antigens. Eight regional controls were tested for peptide binding to all toxin components. The number of samples used for peptide binding and their LT and ET neutralization activities are summarized in Table 5. Magnitude of binding across all decapeptides was uniformly low compared to binding of positive control antibodies (S1 Fig) and compared to sera from human vaccinees [20, 21, 36, 37]. Average IgG binding patterns to PA, LF and EF decapeptides were very similar between serum samples from cutaneous anthrax survivors and regional controls (Fig 5, upper and middle panels). Evaluation of decapeptide binding by cutaneous anthrax survivor IgG in terms of SD above the regional controls revealed no significant (>2SD above controls) common epitopes in the cutaneous group (Fig 5, lower panels). As sera from regional healthy controls lacked LT and ET neutralization activity, while sera from cutaneous anthrax survivors had robust toxin neutralization activity (Table 5), these data indirectly suggest that antibodies recognizing conformational epitopes not detectable by linear fine mapping strategies are responsible for toxin neutralization. Attempted mapping of linear BclA epitopes using 16 cutaneous anthrax survivor sera with BclA ELISA titers ≥80 (S2 Table) also revealed no common linear BclA epitopes (S2 Fig).

**Table 5:** Serum toxin neutralization activities of sample subsets used for epitope mapping.

	LT neutralization	ET neutralization
**Cutaneous cases**		
PA mapping (n = 17)	263.4 ± 92.5	63.7 ± 6.0
LF mapping (n = 10)	394.8 ± 145.2	72.1 ± 8.3
EF mapping (n = 3)	523.2 ± 387.1	90.5 ± 4.7
**Regional controls (n = 8)**^c^	0.13 ± 0.13	18.14 ± 4.32

^a^ED50 ± SEM.

^b^% neutralization at 1:10 dilution ± SEM.

^c^A single panel of eight regional controls was mapped on all three antigens.

## Discussion

While occurrences of natural anthrax infection are very rare in developed countries and are decreasing in endemic regions including Turkey [5], the postal attacks in the United States in 2001 [1] suggest a continued threat of intentional release of anthrax spores. Studying the immune response of individuals who have recovered from anthrax infection provides a unique opportunity to determine how the immune system responds to native antigens during anthrax infection. This information can then be used to inform future treatments and approaches to vaccination. Moreover, serum antibodies from recovered cutaneous anthrax patients provide a record of exposure of the immune system to antigens that are not present in anthrax vaccines given in the western world [28, 38, 39]. While there have been many case reports detailing clinical information on individual patients, fewer studies have examined the cellular [17, 18, 40–42] or humoral [16, 18, 19, 43–49] immune response to cutaneous anthrax infection. The present study is the first to investigate three facets of cutaneous infection: the impact of disease severity on the humoral immune response, the fine specificity of the anti-toxin antibody response, and incidence of the humoral response to BclA and anthrose.

The humoral immune response to toxin, capsule and spore antigens was assessed in 46 cutaneous anthrax survivors from the Kayseri and Malatya regions of Turkey. As expected, antibodies specific to all three anthrax toxin components were present in the recovered cutaneous anthrax serum samples. Interestingly, toxin-binding antibodies were also detected in the sera of some uninfected healthy controls from the region, suggesting that these healthy, Turkish controls may have previously experienced subclinical infection. Evidence for such occurrences have been previously reported [32–35]. Similarly, healthy controls from an anthrax endemic region in India have higher anti-PA and anti-LF values than healthy controls drawn from a non-endemic region [50, 51]. Notably, however, LT and ET neutralizing responses were absent in these samples, as were several correlative relationships between antibody specificities that were observed in the cases. These differing responses may be explained by the subclinical nature of infection from low numbers of spores in the regional control group. Alternative possibilities include antibody binding to inactivated forms of the toxin antigens or the presence of antibodies specific for other antigen(s) that are cross-reactive with the anthrax toxin components.

Cutaneous anthrax infection exposes the immune system to the entire life cycle of *B*. *anthracis*, presenting a different array of antigens than either of the cell-free filtrate vaccines [28, 38, 39]. We confirmed cutaneous anthrax infection generates a humoral immune response to the poly-γ-D-glutamic acid capsule antigen, as well as two different spore antigens in many individuals, demonstrating their immunogenicity in humans. However, mapping of BclA IgG binding to overlapping decapeptides of BclA failed to reveal any common linear epitopes, suggesting that BclA responses in the setting of natural infection may target conformational epitopes. The combination of toxin, capsule and spore-specific antibodies observed in individuals who have recovered from cutaneous anthrax could help to explain why re-infection in humans is extremely rare [4]. In support of this possibility, capsule-specific antibodies have been shown to protect both mice and Rhesus macaques from lethal Ames strain challenge [52, 53]. Further, immunization with BclA in combination with PA provides greater protection than PA alone [54, 55]. While anthrose is currently being investigated for its diagnostic specificity [56, 57], it has not yet been tested as a vaccine candidate. Our findings suggest all three antigens are immunogenic in a significant fraction of individuals after exposure, and should be further tested as potential vaccine components.

While all 46 patients in this study had previously been infected with cutaneous anthrax, the severity of the infection varied person to person. The samples were classified as mild or severe using previously established criteria [5]. We observed that patients with severe cutaneous anthrax had a more robust humoral immune response than those with a mild disease course, resulting in not only increased PA IgG concentrations and LF IgG titers, but also increased LT and ET neutralization. While anthrax toxins are thought to be immunosuppressive [14, 15], our findings suggest that a greater adaptive immune response is generated in severe disease, perhaps due to exposure to higher levels of antigen over longer periods of time.

Investigating the fine specificity of the humoral response to cutaneous anthrax infection could result in the discovery of particular immune targets that correlate with toxin neutralization. However, sequential epitope binding to PA, LF and EF revealed no differences in the magnitude or pattern of binding to decapeptides between recovered patients and controls. As only sera from the recovered patients neutralized either LT or ET, we reason that the toxin neutralizing antibody response in these samples is likely primarily directed to conformational epitopes.

There are several limitations to this study that should be noted. An important limitation is that the humoral responses measured in this study cannot be correlated with protection. First, antibiotic therapy, not the natural response to infection, was used to cure the cutaneous anthrax survivors. Second, humoral protection can be afforded by mechanisms other than toxin neutralization, including bacterial or spore clearance. Indeed, the antibodies to capsule, anthrose and BclA detected in this study may contribute to protection by these mechanisms. Third, vaccinated animals lacking detectable neutralizing antibodies can be protected after lethal challenge, coincident with boosted humoral responses [58]. The possibility that some of the regional controls in the present study could mount such responses upon challenge cannot be discounted. Finally, our method of measuring toxin neutralization, though standard in the field, may have missed neutralizing antibodies that may act by blocking LF/EF binding sites on multimerized PA at the cell surface [59].

We conclude that cutaneous anthrax infection elicits antibodies to all three anthrax toxin components, as well as capsule and spore antigens, that severe infection increases rather than decreases the humoral immune response in the context of antibiotic therapy, and that the toxin neutralizing antibodies in cutaneous anthrax survivors are very likely directed to conformational epitopes. Although vaccination can raise toxin-neutralizing antibodies that recognize linear epitopes [20], our results support the production of vaccine formulations that preserve native antigen structures that mimic those encountered in the context of natural infection.

## Supporting information

S1 Fig Quality control assessment of solid phase, overlapping peptides of *bacillus anthracis* toxin antigens. Solid phase, overlapping peptides of Protective Antigen (PA, Panel **A**), Lethal Factor (LF, Panel **B**) and Edema Factor (EF, Panel **C**) were quality control tested using positive control samples (upper panels), a human negative control reference serum (middle panels), and HRP-conjugated anti-human IgG secondary antibody alone (lower panels). Sources and dilutions of positive control antibodies are as follows: Panel A: List Biologicals PA polyclonal antibody 771b tested at 0.25 μg/well; Panel B: LF positive human Anthrax Vaccine Absorbed sample 560006 from Crowe, et al. Vaccine 29:3670–3678, 2011 tested at 1:200 dilution; Panel C: List Biologicals EF polyclonal antibody 7732a2 tested at 0.5 μg/well.
(TIF)

S2 Fig Binding of case and regional control serum IgG to overlapping decapeptides of BclA. **(A)** Average IgG binding to overlapping decapeptides of *Bacillus* collagen like protein of *anthracis* (BclA) using serum samples from 16 recovered cutaneous anthrax cases with BclA IgG titers ≥ 80 (top) and 8 regional Turkish controls (middle). The lower panel shows binding of sera from the 16 BclA IgG positive cases above the mean of the 8 regional controls expressed as number of SD above the mean for each decapeptide. **(B)** Average IgG binding to overlapping decapeptides of BclA using a positive control rabbit anti-BclA antibody (pAb NR9578, BEI Resources; top), human negative control reference serum sample 510051 (middle), and HRP-conjugated anti-rabbit secondary antibody alone (lower).
(TIF)

S1 Table Cutaneous anthrax patient information. (PDF)

S2 Table Serology values, toxin neutralization values, and samples used in epitope mapping studies. (PDF)

## References

[1] Jernigan JA, Stephens DS, Ashford DA, Omenaca C, Topiel MS, Galbraith M, et al Bioterrorism-related inhalational anthrax: the first 10 cases reported in the United States. Emerg Infect Dis. 2001;7(6):933–44. 10.3201/eid0706.010604 11747719 PMC2631903

[2] Swartz MN. Recognition and management of anthrax—an update. N Engl J Med. 2001;345(22):1621–6. 10.1056/NEJMra012892 11704686

[3] Berger T, Kassirer M, Aran AA. Injectional anthrax—new presentation of an old disease. Euro Surveill. 2014;19(32). 10.2807/1560-7917.es2014.19.32.20877 25139073

[4] Anthrax in Humans and Animals. WHO Guidelines Approved by the Guidelines Review Committee. 4th ed. Geneva 2008.

[5] Doganay M, Metan G, Alp E. A review of cutaneous anthrax and its outcome. J Infect Public Health. 2010;3(3):98–105. 10.1016/j.jiph.2010.07.004 20869669

[6] Dixon TC, Meselson M, Guillemin J, Hanna PC. Anthrax. N Engl J Med. 1999;341(11):815–26. 10.1056/NEJM199909093411107 10477781

[7] Mock M, Fouet A. Anthrax. Annu Rev Microbiol. 2001;55:647–71. 10.1146/annurev.micro.55.1.647 11544370

[8] Zwartouw HT, Smith H. Polyglutamic acid from Bacillus anthracis grown in vivo; structure and aggressin activity. Biochem J. 1956;63(3):437–42. 10.1042/bj0630437 13341899 PMC1216191

[9] Stanley JL, Smith H. Purification of factor I and recognition of a third factor of the anthrax toxin. J Gen Microbiol. 1961;26:49–63. 10.1099/00221287-26-1-49 13916257

[10] Stanley JL, Sargeant K, Smith H. Purification of factors I and II of the anthrax toxin produced in vivo. J Gen Microbiol. 1960;22:206–18. 10.1099/00221287-22-1-206 13833788

[11] Bradley KA, Mogridge J, Mourez M, Collier RJ, Young JA. Identification of the cellular receptor for anthrax toxin. Nature. 2001;414(6860):225–9. 10.1038/n35101999 11700562

[12] Klimpel KR, Arora N, Leppla SH. Anthrax toxin lethal factor contains a zinc metalloprotease consensus sequence which is required for lethal toxin activity. Mol Microbiol. 1994;13(6):1093–100. 10.1111/j.1365-2958.1994.tb00500.x 7854123

[13] Leppla SH. Anthrax toxin edema factor: a bacterial adenylate cyclase that increases cyclic AMP concentrations of eukaryotic cells. Proc Natl Acad Sci U S A. 1982;79(10):3162–6. 10.1073/pnas.79.10.3162 6285339 PMC346374

[14] Tournier JN, Rossi Paccani S, Quesnel-Hellmann A, Baldari CT. Anthrax toxins: a weapon to systematically dismantle the host immune defenses. Mol Aspects Med. 2009;30(6):456–66. 10.1016/j.mam.2009.06.002 19560486

[15] Moayeri M, Leppla SH, Vrentas C, Pomerantsev AP, Liu S. Anthrax Pathogenesis. Annu Rev Microbiol. 2015;69:185–208. 10.1146/annurev-micro-091014-104523 26195305

[16] Brenneman KE, Doganay M, Akmal A, Goldman S, Galloway DR, Mateczun AJ, et al The early humoral immune response to Bacillus anthracis toxins in patients infected with cutaneous anthrax. FEMS Immunol Med Microbiol. 2011;62(2):164–72. 10.1111/j.1574-695X.2011.00800.x 21401726 PMC3605738

[17] Ingram RJ, Metan G, Maillere B, Doganay M, Ozkul Y, Kim LU, et al Natural exposure to cutaneous anthrax gives long-lasting T cell immunity encompassing infection-specific epitopes. J Immunol. 2010;184(7):3814–21. 10.4049/jimmunol.0901581 20208010

[18] Laws TR, Kuchuloria T, Chitadze N, Little SF, Webster WM, Debes AK, et al A Comparison of the Adaptive Immune Response between Recovered Anthrax Patients and Individuals Receiving Three Different Anthrax Vaccines. PLoS One. 2016;11(3):e0148713 10.1371/journal.pone.0148713 27007118 PMC4805272

[19] Boyer AE, Quinn CP, Beesley CA, Gallegos-Candela M, Marston CK, Cronin LX, et al Lethal factor toxemia and anti-protective antigen antibody activity in naturally acquired cutaneous anthrax. J Infect Dis. 2011;204(9):1321–7. 10.1093/infdis/jir543 21908727 PMC3182309

[20] Crowe SR, Ash LL, Engler RJ, Ballard JD, Harley JB, Farris AD, et al Select human anthrax protective antigen epitope-specific antibodies provide protection from lethal toxin challenge. J Infect Dis. 2010;202(2):251–60. 10.1086/653495 20533877 PMC2891133

[21] Crowe SR, Garman L, Engler RJ, Farris AD, Ballard JD, Harley JB, et al Anthrax vaccination induced anti-lethal factor IgG: fine specificity and neutralizing capacity. Vaccine. 2011;29(20):3670–8. 10.1016/j.vaccine.2011.03.011 21420416 PMC3233230

[22] Smith K, Crowe SR, Garman L, Guthridge CJ, Muther JJ, McKee E, et al Human monoclonal antibodies generated following vaccination with AVA provide neutralization by blocking furin cleavage but not by preventing oligomerization. Vaccine. 2012;30(28):4276–83. 10.1016/j.vaccine.2012.03.002 22425791 PMC3367042

[23] Duman EK, Gross T, Larabee J, Pate L, Cuthbertson H, Charlton S, et al Anthrax Vaccine Precipitated Induces Edema Toxin-Neutralizing, Edema Factor-specific antibodies in human recipients. Clin Vaccine Immunol. 2017;in press (CVI00165-17R1). 10.1128/CVI.00165-17 PMC5674197 28877928

[24] Quinn CP, Semenova VA, Elie CM, Romero-Steiner S, Greene C, Li H, et al Specific, sensitive, and quantitative enzyme-linked immunosorbent assay for human immunoglobulin G antibodies to anthrax toxin protective antigen. Emerg Infect Dis. 2002;8(10):1103–10. 10.3201/eid0810.020380 12396924 PMC2730307

[25] Brahmbhatt TN, Darnell SC, Carvalho HM, Sanz P, Kang TJ, Bull RL, et al Recombinant exosporium protein BclA of Bacillus anthracis is effective as a booster for mice primed with suboptimal amounts of protective antigen. Infection and immunity. 2007;75(11):5240–7. 10.1128/IAI.00884-07 17785478 PMC2168312

[26] Mehta AS, Saile E, Zhong W, Buskas T, Carlson R, Kannenberg E, et al Synthesis and antigenic analysis of the BclA glycoprotein oligosaccharide from the Bacillus anthracis exosporium. Chemistry. 2006;12(36):9136–49. 10.1002/chem.200601245 17133642

[27] Saile E, Boons GJ, Buskas T, Carlson RW, Kannenberg EL, Barr JR, et al Antibody responses to a spore carbohydrate antigen as a marker of nonfatal inhalation anthrax in rhesus macaques. Clin Vaccine Immunol. 2011;18(5):743–8. 10.1128/CVI.00475-10 21389148 PMC3122534

[28] Charlton S, Herbert M, McGlashan J, King A, Jones P, West K, et al A study of the physiology of Bacillus anthracis Sterne during manufacture of the UK acellular anthrax vaccine. J Appl Microbiol. 2007;103(5):1453–60. 10.1111/j.1365-2672.2007.03391.x 17953556

[29] Li H, Soroka SD, Taylor TH Jr., Stamey KL, Stinson KW, Freeman AE, et al Standardized, mathematical model-based and validated in vitro analysis of anthrax lethal toxin neutralization. J Immunol Methods. 2008;333(1–2):89–106. 10.1016/j.jim.2008.01.007 18304568

[30] Larabee JL, Maldonado-Arocho FJ, Pacheco S, France B, DeGiusti K, Shakir SM, et al Glycogen synthase kinase 3 activation is important for anthrax edema toxin-induced dendritic cell maturation and anthrax toxin receptor 2 expression in macrophages. Infect Immun. 2011;79(8):3302–8. 10.1128/IAI.05070-11 21576335 PMC3147554

[31] James JA, Scofield RH, Harley JB. Basic amino acids predominate in the sequential autoantigenic determinants of the small nuclear 70K ribonucleoprotein. Scand J Immunol. 1994;39(6):557–66. 10.1111/j.1365-3083.1994.tb03413.x 7516572

[32] Norman PS, Ray JG Jr., Brachman PS, Plotkin SA, Pagano JS. Serologic testing for anthrax antibodies in workers in a goat hair processing mill. Am J Hyg. 1960;72:32–7. 10.1093/oxfordjournals.aje.a120132 14427621

[33] Wattiau P, Govaerts M, Frangoulidis D, Fretin D, Kissling E, Van Hessche M, et al Immunologic response of unvaccinated workers exposed to anthrax, Belgium. Emerg Infect Dis. 2009;15(10):1637–40. 10.3201/eid1510.081717 19861061 PMC2866386

[34] Kissling E, Wattiau P, China B, Poncin M, Fretin D, Pirenne Y, et al B. anthracis in a wool-processing factory: seroprevalence and occupational risk. Epidemiol Infect. 2012;140(5):879–86. 10.1017/S0950268811001488 21835070

[35] Doolan DL, Freilich DA, Brice GT, Burgess TH, Berzins MP, Bull RL, et al The US capitol bioterrorism anthrax exposures: clinical epidemiological and immunological characteristics. J Infect Dis. 2007;195(2):174–84. 10.1086/510312 17191162

[36] Dumas EK, Garman L, Cuthbertson H, Charlton S, Hallis B, Engler RJM, et al Lethal factor antibodies contribute to lethal toxin neutralization in recipients of anthrax vaccine precipitated. Vaccine. 2017;35(26):3416–22. 10.1016/j.vaccine.2017.05.006 28504191 PMC5512426

[37] Dumas EK, Gross T, Larabee J, Pate L, Cuthbertson H, Charlton S, et al Anthrax Vaccine Precipitated Induces Edema Toxin-Neutralizing, Edema Factor-Specific Antibodies in Human Recipients. Clin Vaccine Immunol. 2017;24(11). 10.1128/CVI.00165-17 PMC5674197 28877928

[38] Turnbull PC. Anthrax vaccines: past, present and future. Vaccine. 1991;9(8):533–9. 10.1016/0264-410x(91)90237-z 1771966

[39] Wright JG, Quinn CP, Shadomy S, Messonnier N. Use of anthrax vaccine in the United States: recommendations of the Advisory Committee on Immunization Practices (ACIP), 2009. MMWR Recomm Rep. 2010;59(RR-6):1–30. 20651644

[40] Ingram RJ, Harris A, Ascough S, Metan G, Doganay M, Ballie L, et al Exposure to anthrax toxin alters human leucocyte expression of anthrax toxin receptor 1. Clin Exp Immunol. 2013;173(1):84–91. 10.1111/cei.12090 23607659 PMC3694538

[41] Ingram RJ, Ascough S, Reynolds CJ, Metan G, Doganay M, Baillie L, et al Natural cutaneous anthrax infection, but not vaccination, induces a CD4(+) T cell response involving diverse cytokines. Cell Biosci. 2015;5:20 10.1186/s13578-015-0011-4 26075052 PMC4464127

[42] Ascough S, Ingram RJ, Chu KK, Reynolds CJ, Musson JA, Doganay M, et al Anthrax lethal factor as an immune target in humans and transgenic mice and the impact of HLA polymorphism on CD4+ T cell immunity. PLoS Pathog. 2014;10(5):e1004085 10.1371/journal.ppat.1004085 24788397 PMC4006929

[43] Turnbull PC, Broster MG, Carman JA, Manchee RJ, Melling J. Development of antibodies to protective antigen and lethal factor components of anthrax toxin in humans and guinea pigs and their relevance to protective immunity. Infection and immunity. 1986;52(2):356–63. 3084381 10.1128/iai.52.2.356-363.1986 PMC261006

[44] Turnbull PC, Leppla SH, Broster MG, Quinn CP, Melling J. Antibodies to anthrax toxin in humans and guinea pigs and their relevance to protective immunity. Med Microbiol Immunol (Berl). 1988;177(5):293–303. 3139974 10.1007/BF00189414

[45] Semenova VA, Schmidt DS, Taylor TH Jr., Li H, Steward-Clark E, Soroka SD, et al Analysis of anti-protective antigen IgG subclass distribution in recipients of anthrax vaccine adsorbed (AVA) and patients with cutaneous and inhalation anthrax. Vaccine. 2007;25(10):1780–8. 10.1016/j.vaccine.2006.11.028 17229495

[46] Sirisanthana T, Nelson KE, Ezzell JW, Abshire TG. Serological studies of patients with cutaneous and oral-oropharyngeal anthrax from northern Thailand. Am J Trop Med Hyg. 1988;39(6):575–81. 10.4269/ajtmh.1988.39.575 3144920

[47] Harrison LH, Ezzell JW, Abshire TG, Kidd S, Kaufmann AF. Evaluation of serologic tests for diagnosis of anthrax after an outbreak of cutaneous anthrax in Paraguay. J Infect Dis. 1989;160(4):706–10. 10.1093/infdis/160.4.706 2507648

[48] de Lalla F, Ezzell JW, Pellizzer G, Parenti E, Vaglia A, Marranconi F, et al Familial outbreak of agricultural anthrax in an area of northern Italy. Eur J Clin Microbiol Infect Dis. 1992;11(9):839–42. 10.1007/bf01960887 1468425

[49] Quinn CP, Dull PM, Semenova V, Li H, Crotty S, Taylor TH, et al Immune responses to Bacillus anthracis protective antigen in patients with bioterrorism-related cutaneous or inhalation anthrax. J Infect Dis. 2004;190(7):1228–36. 10.1086/423937 15346332

[50] Ghosh N, Goel AK. Anti-protective antigen IgG enzyme-linked immunosorbent assay for diagnosis of cutaneous anthrax in India. Clin Vaccine Immunol. 2012;19(8):1238–42. 10.1128/CVI.00154-12 22718130 PMC3416091

[51] Ghosh N, Tomar I, Lukka H, Goel AK. Serodiagnosis of human cutaneous anthrax in India using an indirect anti-lethal factor IgG enzyme-linked immunosorbent assay. Clin Vaccine Immunol. 2013;20(2):282–6. 10.1128/CVI.00598-12 23269414 PMC3571271

[52] Joyce J, Cook J, Chabot D, Hepler R, Shoop W, Xu Q, et al Immunogenicity and protective efficacy of Bacillus anthracis poly-gamma-D-glutamic acid capsule covalently coupled to a protein carrier using a novel triazine-based conjugation strategy. The Journal of biological chemistry. 2006;281(8):4831–43. 10.1074/jbc.M509432200 16293624

[53] Chabot DJ, Ribot WJ, Joyce J, Cook J, Hepler R, Nahas D, et al Protection of rhesus macaques against inhalational anthrax with a Bacillus anthracis capsule conjugate vaccine. Vaccine. 2016;34(34):4012–6. 10.1016/j.vaccine.2016.06.031 27329184

[54] Cote CK, Kaatz L, Reinhardt J, Bozue J, Tobery SA, Bassett AD, et al Characterization of a multi-component anthrax vaccine designed to target the initial stages of infection as well as toxaemia. J Med Microbiol. 2012;61(Pt 10):1380–92. 10.1099/jmm.0.045393-0 22767539 PMC3541767

[55] Kohler SM, Baillie LW, Beyer W. BclA and toxin antigens augment each other to protect NMRI mice from lethal Bacillus anthracis challenge. Vaccine. 2015;33(24):2771–7. 10.1016/j.vaccine.2015.04.049 25917676

[56] Tamborrini M, Oberli MA, Werz DB, Schurch N, Frey J, Seeberger PH, et al Immuno-detection of anthrose containing tetrasaccharide in the exosporium of Bacillus anthracis and Bacillus cereus strains. J Appl Microbiol. 2009;106(5):1618–28. 10.1111/j.1365-2672.2008.04129.x 19226390

[57] Dhenin SG, Moreau V, Nevers MC, Creminon C, Djedaini-Pilard F. Sensitive and specific enzyme immunoassays for antigenic trisaccharide from Bacillus anthracis spores. Org Biomol Chem. 2009;7(24):5184–99. 10.1039/b914534f 20024115

[58] Chen L, Schiffer JM, Dalton S, Sabourin CL, Niemuth NA, Plikaytis BD, et al Comprehensive analysis and selection of anthrax vaccine adsorbed immune correlates of protection in rhesus macaques. Clin Vaccine Immunol. 2014;21(11):1512–20. 10.1128/CVI.00469-14 25185577 PMC4248764

[59] Clement KH, Rudge TL Jr., Mayfield HJ, Carlton LA, Hester A, Niemuth NA, et al Vaccination of rhesus macaques with the anthrax vaccine adsorbed vaccine produces a serum antibody response that effectively neutralizes receptor-bound protective antigen in vitro. Clin Vaccine Immunol. 2010;17(11):1753–62. 10.1128/CVI.00174-10 20739500 PMC2976102

