# Supplementary figures and images for: Toxin-neutralizing antibodies elicited by naturally acquired cutaneous anthrax are elevated following severe disease and appear to target conformational epitopes
Source: PLoS One. 2020 Apr 15;15(4):e0230782. doi: 10.1371/journal.pone.0230782 (PMC7159215; doi:10.1371/journal.pone.0230782)

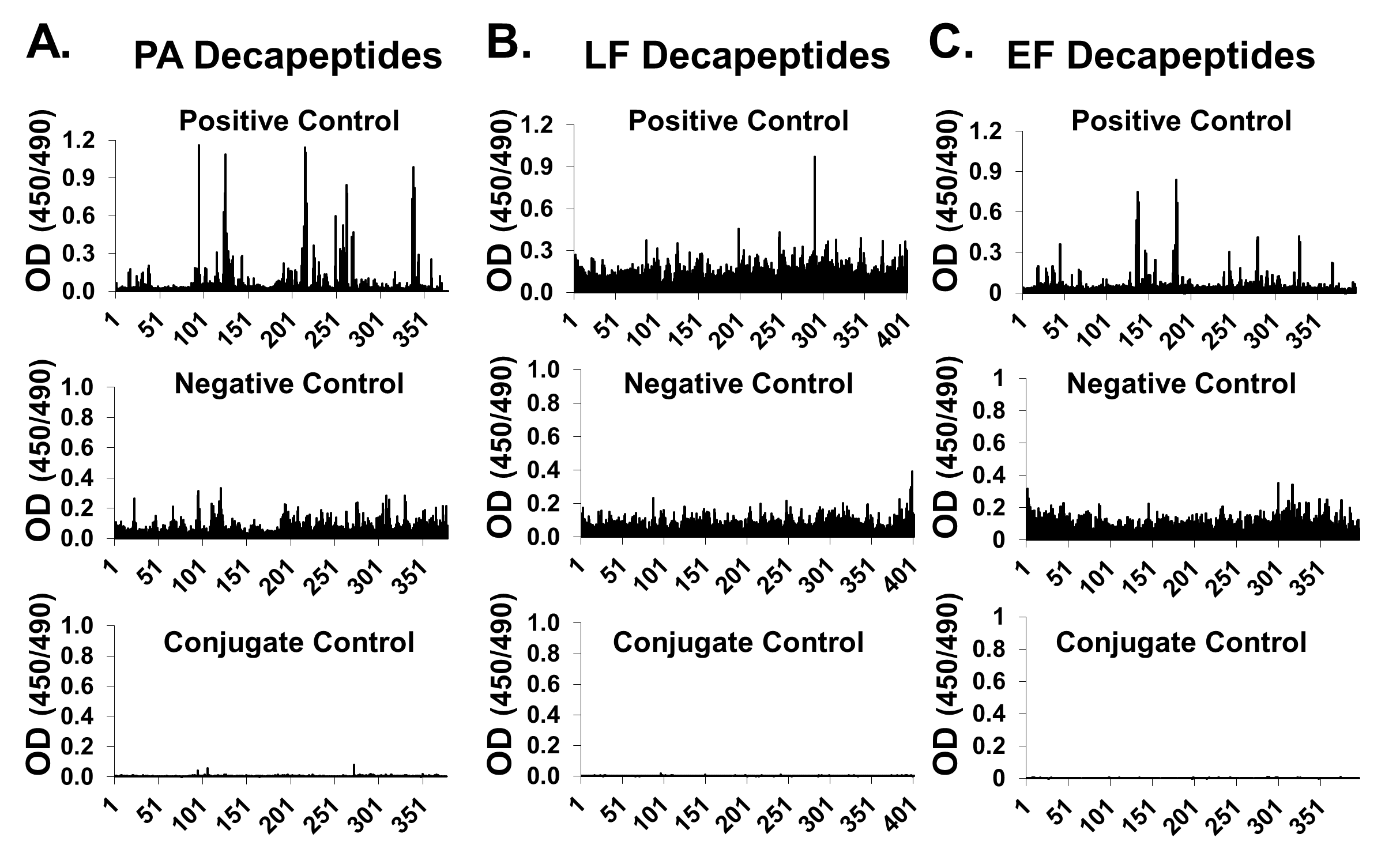

Supplement: S1 Fig — Solid phase, overlapping peptides of Protective Antigen (PA, Panel A), Lethal Factor (LF, Panel B) and Edema Factor (EF, Panel C) were quality control tested using positive control samples (upper panels), a human negative control reference serum (middle panels), and HRP-conjugated anti-human IgG secondary antibody alone (lower panels). Sources and dilutions of positive control antibodies are as follows: Panel A: List Biologicals PA polyclonal antibody 771b tested at 0.25 μg/well; Panel B: LF positive human Anthrax Vaccine Absorbed sample 560006 from Crowe, et al. Vaccine 29:3670–3678, 2011 tested at 1:200 dilution; Panel C: List Biologicals EF polyclonal antibody 7732a2 tested at 0.5 μg/well. (TIF) [file pone.0230782.s001.tif]

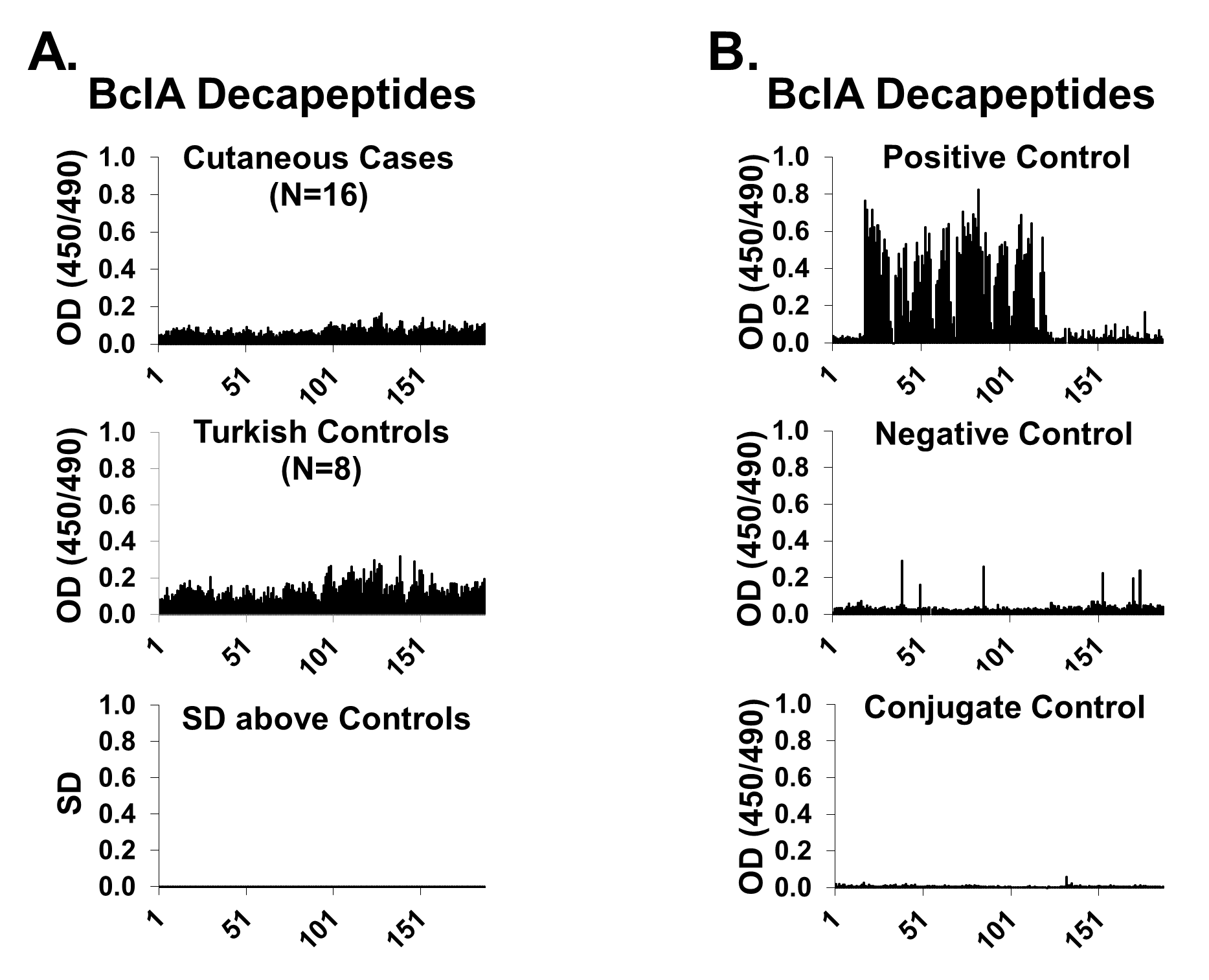

Supplement: S2 Fig — (A) Average IgG binding to overlapping decapeptides of Bacillus collagen like protein of anthracis (BclA) using serum samples from 16 recovered cutaneous anthrax cases with BclA IgG titers ≥ 80 (top) and 8 regional Turkish controls (middle). The lower panel shows binding of sera from the 16 BclA IgG positive cases above the mean of the 8 regional controls expressed as number of SD above the mean for each decapeptide. (B) Average IgG binding to overlapping decapeptides of BclA using a positive control rabbit anti-BclA antibody (pAb NR9578, BEI Resources; top), human negative control reference serum sample 510051 (middle), and HRP-conjugated anti-rabbit secondary antibody alone (lower). (TIF) [file pone.0230782.s002.tif]
