# Supplementary material for: Toxin-neutralizing antibodies elicited by naturally acquired cutaneous anthrax are elevated following severe disease and appear to target conformational epitopes
Source: PLoS One. 2020 Apr 15;15(4):e0230782. doi: 10.1371/journal.pone.0230782 (PMC7159215; doi:10.1371/journal.pone.0230782)
Supplement: S1 Table — (PDF) [file pone.0230782.s003.pdf]

S1 Table. Cutaneous anthrax patient information

Patient no.	Age	Gender	Infection Date[a]	Draw Date[a]	Incubation (d)	Time post infection[b] (m)	Contact with contaminated animal materials	Lesion site	Severity of infection	Gram stain	Culture	Previous antibiotic use	Antibiotic therapy[f]	Duration of therapy (d)	Outcome
1	24	M	2006	2007	5	17.2	+	Right arm	Mild	-	-	Unknown	Amox	5	recovered
2	48	M	2007	2007	7	6.9	+	Left hand	Severe	-	-	Yes	Pen G	10	recovered
3	33	M	2005	2007	6	29.9	+	Both arms	Severe	-	-	Yes	Pen G	5	left deep tissue scar, skin graft
4	23	M	2005	2007	12	33.8	+	Left arm	Mild	-	+	No	Amox	5	recovered
5	19	M	2007	2007	9	4.3	+	Right wrist	Severe	-	-	Yes	Pen G	5	recovered
6	30	M	2003	2007	1	54.2	+	Anterior neck	Severe[c]	+	-	No	Pen G	7	S.aureus bacteremia, recovered
7	30	M	2007	2007	9	4.3	+	Left wrist	Mild	-	-	No	Dox	5	recovered
8	55	F	2005	2007	6	30.5	+	Left hand finger	Mild	+	-	Yes	Amox	5	recovered
9	33	M	2005	2007	6	89.8	+	Left hand and wrist	Severe	+	-	Unknown	Clind	5	left deep tissue scar, skin graft
10	18	F	2005	2007	15	30.4	+	Left arm	Mild	-	-	Yes	Amox	3	recovered
11	23	M	2006	2012	Unknown	66.8	+	Right arm	Severe	-	-	Yes	Pen G	5	Recovered
12	39	M	2007	2012	12	51.2	+	Right hand	Mild	+	+	No	Pen G	5	Recovered
13	52	F	2007	2012	5	53.6	+	Left hand finger	Mild	-	-	Yes	Pro Pen	Unknown	Recovered
14	30	M	2007	2012	2	53.5	+	Left wrist	Severe	-	-	No	Pen G	10	Recovered
15	21	F	2008	2012	2	42.1	+	Right arm	Severe	-	-	No	Cipro	5	Recovered
16	27	M	2008	2012	20	42.5	+	Right arm	Severe	-	-	Yes	Pen G	5	Recovered
17	20	F	2010	2012	6	22.9	+	Left wrist	Mild	-	-	No	Dox	5	Recovered
18	48	F	2010	2012	3	22.9	+	Right hand finger	Mild	+	-	No	Pro Pen	5	Recovered
19	16	M	2010	2012	3	22.9	+	Left hand finger	Mild	+	-	No	Dox	5	Recovered
20	54	M	2011	2012	6	7.6	+	Right arm	Severe	-	-	No	Pen G	8	Recovered
21	23	M	2011	2012	2	5.3	+	Left wrist and hand	Mild	-	+	No	SAM	5	Recovered
22	23	M	2011	2012	6	3.9	+	Right hand and elbow	Severe	-	-	No	Pen G	5	Recovered
23	53	F	2012	2012	1	1	+	Right hand	Severe	+	-	No	SAM	Unknown	Recovered
24	23	F	2004	2012	Unknown	95.1	Unknown	Left face	Severe			No	Pen G	5	Recovered
25	26	M	2010	2012	Unknown	17.4	+	Left face	Severe	-	-	No	Pen G	5	Recovered
26	37	M	2004	2012	4	102.2	+	Right hand finger	Mild	-	-	No	Pro Pen	7	Recovered
27	42	F	2004	2012	4	102.2	+	Right hand finger	Severe	-	-	No	Pro Pen	7	Recovered
28	54	M	2004	2012	4	102.2	+	Both hands fingers	Severe	-	-	Yes	Pro Pen	7	Recovered
29	27	M	2005	2012	3	88.7	+	Left arm and hand	Severe	-	-	No	Pen G	5	Recovered
30	43	M	2006	2012	Unknown	77.8	-	Right arm and left neck	Severe[d]	-	-	Yes	Pen G	14	Recovered
31	63	F	2005	2012	4	88.8	+	Both hands fingers	Mild	+	+	No	Pen G	7	Recovered
32	45	F	2005	2012	4	88.9	+	Left eyelid	Severe[e]	+	+	No	Pen G+Cipro	14	Recovered
33	23	F	2007	2012	6	66.2	+	Right wrist	Severe	-	-	Yes	Pen G	7	Recovered
34	22	M	2008	2012	4	49.8	+	Both hands fingers	Severe	+	+	No	Amox	4	Recovered
35	51	M	2008	2012	6	49.7	+	Right hand fingers	Mild	+	+	No	Pro Pen	4	Recovered
36	33	M	2006	2012	2	77.8	+	Right hand finger	Mild	-	-	No	Pro Pen	3	Recovered
37	18	M	2009	2012	4	42.3	+	Right arm and finger	Mild	+	-	No	Amox	7	Recovered
38	52	M	2012	2012	7	3.5	+	Left hand	Severe	+	-	Yes	Pen G	5	Recovered
39	38	M	2008	2012	5	49.8	+	Right arm	Severe	-	-	Yes	Pen G	7	Recovered
40	44	M	2008	2012	Unknown	52.8	+	Left arm	Severe	-	-	Yes	Pro Pen	7	Recovered
41	16	M	2005	2012	5	88.5	+	Left hand finger	Mild	-	-	No	Pen G	5	Recovered
42	48	M	2005	2012	5	88.5	+	Right wrist and hand	Severe	+	+	No	Pen G	7	Recovered
43	57	M	2005	2012	3	88.4	+	Right arm and wrist	Mild	-	-	No	Pen G	5	Recovered
44	40	F	2012	2012	4	3.2	+	Left hand fingers	Severe	+	+	No	Pen G+Amox	8	Recovered
45	48	M	2008	2012	4	51.5	+	Right hand fingers	Severe	+	+	No	Pen G	5	Recovered
46	53	M	2012	2012	Unknown	2.7	-	Left arm	Severe	-	-	Yes	SAM	5	Recovered

[a] Only years are provided to protect patient identity
[b] Time in months between infection and blood draw
[c] The patient developed toxemic shock with extensive edema
[d] The patient developed toxemic shock with malignant edema
[e] The patient developed malignant edema
[f] Amox=amoxicillin; Pen G=penicillin G; Dox=doxycycline; Clind=clindamycin; Pro Pen=procaine penicillin; Cipro=Ciprofloxacin
