# Supplementary material for: Toxin-neutralizing antibodies elicited by naturally acquired cutaneous anthrax are elevated following severe disease and appear to target conformational epitopes
Source: PLoS One. 2020 Apr 15;15(4):e0230782. doi: 10.1371/journal.pone.0230782 (PMC7159215; doi:10.1371/journal.pone.0230782)
Supplement: S2 Table — (PDF) [file pone.0230782.s004.pdf]

Table S2. Serology values, toxin neutralization values, and samples used in epitope mapping studies

Sample Number [a]	PA IgG (mg/mL)	PA IgG Titer [b]	LF IgG Titer [b]	EF IgG Titer [b]	Capsule IgG Titer [b,c]	Anthrose IgG Titer [b]	BclA IgG Titer [b]	LT TNA ED50	ET % Neut 1:10	ET % Neut 1:100	PA mapping [d]	LF mapping [d]	EF mapping [d]	BclA Mapping[d]
1	17.20	100.00	100.00	Neg	N/A	Neg	100.00	80.29	64.90	23.10	✓	✓		✓
2	8.90	10.00	10.00	Neg	N/A	Neg	100.00	67.37	61.12	25.52				✓
3	6.10	100.00	Neg	Neg	N/A	Neg	100.00	49.83	22.95	0.00	✓			✓
4	7.70	100.00	10.00	Neg	N/A	Neg	100.00	65.03	44.53	16.63	✓			✓
5	16.10	100.00	100.00	Neg	N/A	Neg	100.00	211.20	77.34	42.70	✓	✓		✓
6	16.10	100.00	Neg	Neg	N/A	Neg	1000.00	147.40	53.01	17.37	✓			✓
7	31.10	100.00	Neg	Neg	N/A	Neg	100.00	38.65	53.56	11.46	✓			✓
8	10.70	100.00	Neg	Neg	N/A	Neg	100.00	21.63	13.86	0.43	✓			✓
9	18.80	100.00	100.00	Neg	N/A	Neg	100.00	252.40	78.93	48.43	✓	✓		✓
10	1.80	Neg	Neg	Neg	N/A	Neg	100.00	16.69	22.75	3.77				✓
11	15.29	80.00	10.00	Neg	Neg	Neg	Neg	134.40	78.20	21.81	✓			
12	2.10	Neg	40.00	Neg	Neg	Neg	320.00	3.07	4.37	0.87				✓
13	2.43	Neg	1280.00	Neg	640.00	Neg	2560.00	17.93	16.16	2.49		✓		✓
14	3.05	Neg	40.00	Neg	Neg	Neg	Neg	102.36	16.83	1.41				
15	12.98	Neg	40.00	Neg	Neg	Neg	80.00	195.63	24.22	6.80				✓
16	9.93	Neg	Neg	Neg	40.00	Neg	Neg	29.02	29.88	6.70				
17	25.04	80.00	Neg	Neg	Neg	Neg	Neg	52.73	57.22	21.11	✓			
18	12.41	80.00	Neg	Neg	Neg	80.00	Neg	38.67	54.42	20.16	✓			
19	10.18	Neg	Neg	Neg	80.00	Neg	Neg	28.10	44.52	12.79				
20	15.25	80.00	640.00	Neg	Neg	80.00	320.00	229.78	36.59	11.52	✓	✓		✓
21	15.37	Neg	Neg	Neg	40.00	Neg	320.00	113.00	26.28	11.82				✓
22	27.96	160.00	2560.00	Neg	160.00	640.00	160.00	400.53	86.33	29.61	✓	✓		✓
23	343.60	2560.00	5120.00	Neg	160.00	Neg	20.00	1185.67	89.37	60.62	✓	✓		
24	0.41	Neg	640.00	Neg	Neg	80.00	40.00	0.00	14.93	11.83				
25	21.46	40.00	640.00	Neg	40.00	640.00	80.00	508.23	65.20	24.65				
26	0.38	Neg	10.00	Neg	Neg	Neg	160.00	0.00	39.04	14.01				
27	23.45	80.00	40.00	Neg	10.00	Neg	40.00	40.49	73.42	27.39				
28	10.45	Neg	40.00	Neg	40.00	160.00	20.00	0.00	19.45	9.24				
29	23.35	80.00	20.00	Neg	Neg	Neg	80.00	10.55	51.46	20.96				
30	20.00	160.00	160.00	Neg	40.00	Neg	Neg	40.80	46.93	20.49				
31	8.68	80.00	40.00	Neg	20.00	Neg	Neg	21.17	19.18	4.93				
32	29.84	320.00	640.00	Neg	40.00	Neg	20.00	194.15	96.35	55.11				
33	1.81	20.00	160.00	Neg	40.00	Neg	Neg	15.13	41.45	12.45				
34	25.53	320.00	320.00	160.00	160.00	Neg	Neg	50.13	81.23	21.48	✓	✓	✓	
35	1.43	80.00	80.00	Neg	80.00	320.00	10.00	3.34	8.76	4.64				
36	10.81	160.00	40.00	Neg	40.00	Neg	Neg	33.55	55.17	17.81				
37	2.56	40.00	80.00	Neg	40.00	Neg	Neg	35.29	32.15	9.07				
38	35.40	160.00	320.00	10.00	Neg	Neg	40.00	295.60	89.96	51.97				
39	1.85	40.00	320.00	80.00	320.00	Neg	Neg	35.65	28.05	1.13				
40	8.95	80.00	80.00	Neg	80.00	Neg	160.00	31.12	49.62	12.15				
41	0.70	20.00	40.00	40.00	80.00	Neg	10.00	7.54	34.64	9.50				
42	8.82	80.00	160.00	20.00	160.00	Neg	40.00	25.13	61.29	8.92				
43	0.33	Neg	80.00	Neg	Neg	Neg	80.00	0.06	32.57	5.50				
44	62.90	320.00	320.00	160.00	Neg	Neg	Neg	229.10	93.36	55.05	✓	✓	✓	
45	9.28	10.00	160.00	Neg	Neg	160.00	Neg	114.23	25.94	1.53				
46	183.85	640.00	5120.00	40.00	320.00	Neg	40.00	1290.50	96.81	96.00	✓	✓	✓	
Control 1	0.44	Neg	40.00	Neg	20.00	Neg	Neg	0.00	0.00	0.00	✓	✓	✓	✓
Control 2	0.46	Neg	40.00	40.00	40.00	Neg	Neg	0.00	9.39	0.00	✓	✓	✓	✓
Control 3	0.55	Neg	Neg	80.00	Neg	80.00	40.00	0.00	22.28	9.58	✓	✓	✓	✓
Control 4	1.00	40.00	20.00	Neg	Neg	Neg	Neg	0.00	25.39	4.51				
Control 5	10.61	80.00	Neg	Neg	Neg	Neg	Neg	0.00	7.01	9.75				
Control 6	0.18	Neg	10.00	Neg	Neg	Neg	Neg	0.00	29.58	3.99	✓	✓	✓	✓
Control 7	0.52	Neg	40.00	Neg	40.00	Neg	40.00	0.00	10.73	11.14				
Control 8	13.15	40.00	10.00	Neg	Neg	Neg	Neg	0.00	13.04	0.98	✓	✓	✓	✓
Control 9	0.45	10.00	Neg	Neg	Neg	Neg	Neg	0.00	9.13	13.18				
Control 10	0.19	Neg	40.00	Neg	Neg	Neg	Neg	0.00	13.53	0.00				
Control 11	0.25	Neg	Neg	Neg	Neg	Neg	10.00	0.00	27.68	16.73				
Control 12	1.14	10.00	20.00	20.00	Neg	Neg	Neg	1.05	29.04	1.20	✓	✓	✓	✓
Control 13	0.33	Neg	Neg	Neg	Neg	Neg	Neg	0.00	31.32	12.78				
Control 14	0.42	Neg	20.00	Neg	Neg	Neg	80.00	0.00	31.37	8.48				
Control 15	0.34	Neg	Neg	Neg	40.00	Neg	Neg	0.00	33.80	12.67	✓	✓	✓	✓
Control 16	0.44	Neg	10.00	20.00	Neg	Neg	40.00	0.00	8.01	2.07	✓	✓	✓	✓
Control 17	0.79	Neg	Neg	Neg	Neg	Neg	Neg	0.00	7.81	3.78				
Control 18	0.36	Neg	Neg	20.00	Neg	Neg	Neg	0.00	36.36	11.55				
Control 19	0.41	Neg	Neg	Neg	Neg	Neg	Neg	0.00	26.44	14.72				
Control 20	0.40	Neg	10.00	Neg	Neg	Neg	Neg	0.00	11.19	3.13				

[a] Cutaneous cases 1-46 correspond to the same case numbers in Table S1. Controls 1-20 are regional Turkish controls.

[b] End-point titers were calculated as the last serum dilution exceeding a threshold of positive binding equivalent to 2 SD above the mean of the regional controls; Neg = negative

[c] N/A=not tested; insufficient sample available for testing

[d] Sample used in epitope mapping
